# Bimodal spore release heights in the water column enhance local retention and population connectivity of bull kelp, *Nereocystis luetkeana*

**DOI:** 10.1002/ece3.70177

**Published:** 2024-08-13

**Authors:** Nicholas P. Burnett, Aurora M. Ricart, Tallulah Winquist, Alisha M. Saley, Matthew S. Edwards, Brent Hughes, Jason Hodin, Marissa L. Baskett, Brian Gaylord

**Affiliations:** ^1^ Department of Evolution and Ecology University of California, Davis Davis California USA; ^2^ Department of Neurobiology, Physiology, and Behavior University of California, Davis Davis California USA; ^3^ Bodega Marine Laboratory Bodega Bay California USA; ^4^ Institut de Ciències del Mar (ICM‐CSIC) Barcelona Spain; ^5^ Bigelow Laboratory for Ocean Sciences East Boothbay Maine USA; ^6^ Department of Biology San Diego State University San Diego California USA; ^7^ Department of Biology Sonoma State University Rohnert Park California USA; ^8^ Friday Harbor Labs University of Washington Friday Harbor Washington USA; ^9^ Deparment of Environmental Science and Policy University of California, Davis Davis California USA

**Keywords:** bet‐hedging, conservation, dispersal, macroalgae, propagules

## Abstract

Dispersal of reproductive propagules determines recruitment patterns and connectivity among populations and can influence how populations respond to major disturbance events. Dispersal distributions can depend on propagule release strategies. For instance, the bull kelp, *Nereocystis luetkeana*, can release propagules (spores) from two heights in the water column (“bimodal release”): at the water surface, directly from the reproductive tissues (sori) on the kelp's blades, and near the seafloor after the sori abscise and sink through the water column. *N. luetkeana* is a foundation species that occurs from central California to Alaska and is experiencing unprecedented levels of population declines near its southern range limit. We know little of the kelp's dispersal distributions, which could influence population recovery and restoration. Here, we quantify how bimodal spore release heights affect dispersal outcomes based on a numerical model specifically designed for *N. luetkeana*. The model incorporates oceanographic conditions typical of the species' coastal range and kelp biological traits. With bimodal release heights, 34% of spores are predicted to settle within 10 m of the parental alga and 60% are predicted to disperse beyond 100 m. As an annual species, bimodal release heights can facilitate the local regeneration of adults within a source kelp forest while also supporting connectivity among multiple forests within broader bull kelp metapopulations. To leverage this pattern of bimodal spore dispersal in bull kelp restoration management, directing resources toward strategically located focal populations that can seed other ones could amplify the scale of recovery.

## Graphical abstract

We used a numerical dispersal model to show that the distinct ability of the bull kelp, *Nereocystis luetkeana*, to release spores at both the water surface and near the seafloor can result in similar amounts of short‐ and long‐distance dispersal. As a result, the kelp can reinforce the density of source populations while strengthening connectivity with nearby kelp forests.

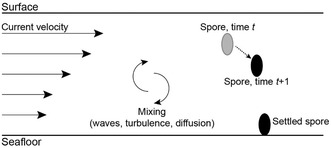

## 1 INTRODUCTION

Dispersal and connectivity among populations are essential for their long‐term persistence. Both properties can shape population structure and resilience following disturbance events such as physical destruction, acute physiological and environmental stress, and disease (Alsuwaiyan et al., 2021; Bernhardt & Leslie, 2013; Breitbach et al., 2012; Dayton et al., 1999; Schleicher et al., 2011; Wernberg et al., 2018). In marine environments, many organisms rely on the fluid‐mediated dispersal of their reproductive propagules (spores, gametes, larvae) whose transport is largely determined by ambient flow conditions (Abelson & Denny, 1997). That is, they are carried from their point of origin by fluid motion, including currents, waves, and turbulence, until they are deposited in a new location. This dispersal strategy is susceptible to temporal and spatial variability in oceanographic conditions, and seemingly leaves little control to the organism itself (Gaylord et al., 2006). However, many species that rely on this dispersal mode exhibit distinct reproductive strategies that can, to some extent, compensate for the stochastic nature of flow‐mediated dispersal (Hodin et al., 2018; Schindler et al., 2015). Example compensating mechanisms include variation in the timing or location of propagule release, especially in response to environmental conditions such as water currents (Edwards & Konar, 2012; Gaylord et al., 2002, 2004; Reed et al., 1997; Scagel, 1947; Schiel & Foster, 2006; Walker, 1980). An additional mechanism is propagule release after the parent organism has been dislodged from the substrate and transported long distances to new areas (Hobday, 2000; Macaya & Zuccarello, 2010).

The stochastic nature of flow‐mediated dispersal is a challenge to understanding long‐distance population connectivity (Cowen & Sponaugle, 2009). Connectivity that subsequently influences population demography depends on whether propagules reach another population while still viable, whether spore settlement at a destination site reaches sufficient densities to produce subsequent life stages (e.g., which can require fusion of gametes), and whether any resultant adults withstand consumption and other environmental stressors. Considering these barriers, it is hypothesized that one successful dispersal strategy in these systems is for the bulk of propagules to be retained near the parent (facilitating persistence of the source population) while the remaining propagules disperse long distances toward other populations (maintaining connectivity) (Amsler & Neushul, 1989; Gadgil, 1971). High levels of local propagule retention can also lead to significant inbreeding, which contributes to reduced genetic diversity and less resistance to environmental variability; long‐distance immigration of propagules can introduce genetic diversity to local populations and limit inbreeding (Gierke et al., 2023). Indeed, this strategy has been observed in some fishes, corals, and seaweeds (Gaylord et al., 2002; Hogan et al., 2012; Schiel & Foster, 2006; Underwood et al., 2006), but it is still unclear how reproductive strategies can affect short‐ versus long‐distance dispersal. Furthermore, dispersal can depend on numerous other biological traits and selective pressures that are not primarily linked to dispersal distance and population connectively (reviewed in Burgess et al., 2016). Once settled, the propagules of some species can become dormant (Carney & Edwards, 2006, 2010; Edwards, 2000; Hoffmann & Santelices, 1991; Klinger, 1985), thereby allowing additional propagules to settle and accrue nearby over time and creating a bank of microscopic stages that are of mixed ages and origins (Carney et al., 2013). This strategy can be especially important to coastal seaweeds, such as kelp, whose microscopic life stages can remain dormant up to several years in some cases, adding to the organisms' range of temporal and spatial dispersal potentials (reviewed in Edwards, 2022).

### 1.1 Kelps use flow‐mediated dispersal

Large seaweeds in the order Laminariales (kelps) are foundation species in marine coastal systems around the world because they form large, complex forests that provide habitat and food to other organisms (Burnett & Koehl, 2018; Graham, 2004; Graham et al., 2007; North, 1971; Schiel & Foster, 2015). They are important drivers of coastal biodiversity (Metzger et al., 2019) and primary productivity (Edwards et al., 2020; Miller et al., 2011; Spector & Edwards, 2020), and they can be important to regulating seawater chemistry (Carrano et al., 2020, 2021; Gonzales et al., 2017) and hydrodynamic flow in the coastal zone (Elsmore et al., 2022, 2023; Gaylord et al., 2007, 2012; Hondolero & Edwards, 2017; Jackson & Winant, 1983). Kelps are typically associated with cold, nutrient‐rich waters, and grow in dense aggregations (forests) that enhance the ecological impact of an individual kelp (Dayton et al., 1999; Schiel & Foster, 2015; Seymour et al., 1989). However, in many locations around the world, kelp forests have declined in recent decades due to adverse hydrographic conditions associated with climate and environmental change (Krumhansl et al., 2016). For instance, ocean warming (Filbee‐Dexter et al., 2016; Filbee‐Dexter & Scheibling, 2014; Smale, 2020), increased frequency and severity of marine heat waves (McPherson et al., 2021; Reed et al., 2016; Rogers‐Bennett & Catton, 2019; Wernberg et al., 2016) and storms (Burnett & Koehl, 2020; Cavanaugh et al., 2011; Dayton et al., 1999; Seymour et al., 1989), climatic events such as the El Niño Southern Oscillation (Edwards, 2004; Edwards et al., 2020; Edwards & Estes, 2006), and overconsumption by grazers (Estes et al., 1998; Jeon et al., 2015; Scheibling et al., 1999) each contribute to the removal and mortality of kelps, although the specific drivers of kelp decline vary geographically. Further, the spatial scales at which these drivers affect kelp populations can differ from the scales at which these populations recover (Edwards, 2004). This mismatch could result from differences in the forcing factors that lead to population losses and population recovery, or from differences in patterns of dispersal and connectivity among kelp populations. For instance, large‐scale changes in environmental conditions, such as during El Niño Southern Oscillation, can lead to widespread population losses, whereas population recovery can rely on small‐scale recolonization of habitat, one kelp forest at a time (reviewed in Edwards, 2022).

Kelps alternate generations between two forms: a macroscopic sporophyte and a microscopic gametophyte (Schiel & Foster, 2006). Declines in kelp populations are usually defined by decreased densities of sporophytes (the life stage that forms kelp forests) due to their conspicuous size, although they are likely influenced by vulnerabilities across their entire life cycle: sporophytes release massive numbers of flagellated spores (up to ~10^12^ spores per individual per year) (Schiel & Foster, 2006), which can remain viable in the water column for several days and disperse with the ambient flow patterns (reviewed in Edwards, 2022). This dispersal can increase with the synchronous release of spores by different individuals (Graham, 2003), which can be greater in specific locations of the forest (Edwards & Konar, 2012), or by the vertical transport of spores into surface waters with wave‐driven longshore currents that can carry them longer distances (Cie & Edwards, 2011). However, spores released near the surface can also experience enhanced grazing pressure by invertebrates such as mysid shrimp, reducing overall settlement (VanMeter & Edwards, 2013). Once settled, spores form either male or female microscopic gametophytes. Male gametophytes release free‐swimming sperm that can locomote only a few millimeters to oogonium that remains attached to the female gametophytes. The union of oogonia and sperm on the female gametophyte produces a diploid zygote that grows into a macroscopic sporophyte (Abbott & Hollenberg, 1976; Coleman et al., 2011; Gaylord et al., 2002, 2006; Schiel & Foster, 2006). Because a minimum density of spores and subsequent gametophytes is required for sperm to find and fertilize oogonia, not all spore dispersal to a given distance within an unoccupied, open habitat results in the appearance of adult sporophytes at that location.

In general, a fragmented or declining kelp forest can recover if spores consistently settle within the spatial extent of the forest, but the origin of spores can vary. For instance, settlement can result from the local retention of propagules (limited dispersal) or from the migration of spores from neighboring populations (longer dispersal), both of which are primarily the outcome of flow‐mediated spore dispersal. In some cases, dispersal can occur as entire sporophytes that dislodge from the substratum form rafts that float long distances to new locations, although spores released from attached adults are generally more numerous than those associated with occasional, dislodged sporophytes (Fraser et al., 2022; Hernández‐Carmona et al., 2006; Hobday, 2000; Layton et al., 2022; Macaya & Zuccarello, 2010). Although the location of spore settlement has a strong role in the location of other life stages, the appearance of sporophytes in that location could still be prevented due to gametophyte mortality from environmental stress, grazing of gametophytes and sporophytes by benthic herbivores (Edwards, 2022; Filbee‐Dexter & Scheibling, 2014; Sala & Graham, 2002; Veenhof, Champion, et al., 2022; Veenhof, Dworjanyn, et al., 2022; Veenhof et al., 2023), or failure of spores to settle in sufficient densities for fertilization of gametes to occur (Allee, 1931; Gascoigne & Lipcius, 2004). Despite these later sources of mortality or failure to fertilize, the spatial distribution of new kelp development begins with spore dispersal patterns. In an era of global kelp forest decline, understanding where mature sporophytes recruit and where entire forests will thrive hinges on a firm grasp of kelp spore dispersal in the water column.

### 1.2 Dominant kelps in California

The bull kelp, *Nereocystis luetkeana* (K. Mertens) Postels & Ruprecht, is a major canopy‐forming kelp ranging from San Luis Obispo County, California to Alaska (Abbott & Hollenberg, 1976). The sporophyte of *N. luetkeana* (hereafter *Nereocystis*) is generally annual and grows a single stipe, up to 36 m long in exceptional cases (Abbott & Hollenberg, 1976), that reaches from the holdfast to the water surface. At the distal end of the stipe is a single large pneumatocyst from which photosynthetic blades grow. Reproductive tissues (sporangia) develop on the blades in distinct patches (sori). When mature, spores are released from the sori. The sori themselves also abscise from the blades over time and sink to the seafloor while still containing viable spores (Walker, 1980). Studies on related species indicate that kelp fragments sink quickly through the water column at speeds up to 0.5 m s^−1^ (Wernberg & Filbee‐Dexter, 2018), suggesting that *Nereocystis* sori spend much longer attached to the blade at the water's surface or resting on the seafloor than they do moving through the water column. Thus, most *Nereocystis* spores are either released from the sori at the surface of the water or near the seafloor after abscission.

The giant kelp, *Macrocystis pyrifera* (Linnaeus) C. Agardh, is also a major canopy‐forming kelp and occurs along most of the western coasts of North and South America, as well as the southern coasts of Africa and Australia (Abbott & Hollenberg, 1976). In contrast to *Nereocystis*, the sporophyte of *M. pyrifera* (hereafter *Macrocystis*) is perennial and grows numerous stipes that reach from the holdfast on the seafloor to the water surface, with photosynthetic blades and air‐filled bladders (pneumatocysts) occurring along the full length of each stipe. The entire thallus can reach lengths up to 45 m (Abbott & Hollenberg, 1976). Sporangia develop on special blades (sporophylls) that occur close to the holdfast and, when mature, release spores into the water column (Gaylord et al., 2002; Reed et al., 1997).

Here, we use numerical simulations to model how the bimodal spore release of *Nereocystis* interacts with ambient flow conditions (including currents, waves, and turbulence) to shape the dispersal distributions of the kelp. We compare these outputs to the existing analysis of unimodal, point release of spores in *Macrocystis* (Gaylord et al., 2002). Populations of *Macrocystis* and *Nereocystis* have declined along the California coast, due in part to severe storms, marine heatwaves, an outbreak of kelp‐grazing sea urchins, and a decline in the natural predators of urchins (Cavanaugh et al., 2011; Dayton et al., 1999; Rogers‐Bennett & Catton, 2019; Schiel & Foster, 2015; Seymour et al., 1989; Tegner et al., 1995). Understanding spore release strategies and dispersal distributions of each species can provide insights into how dispersal might influence population persistence for each of these kelps.

## 2 METHODS

### 2.1 Overview of the dispersal model

**FIGURE 1:**
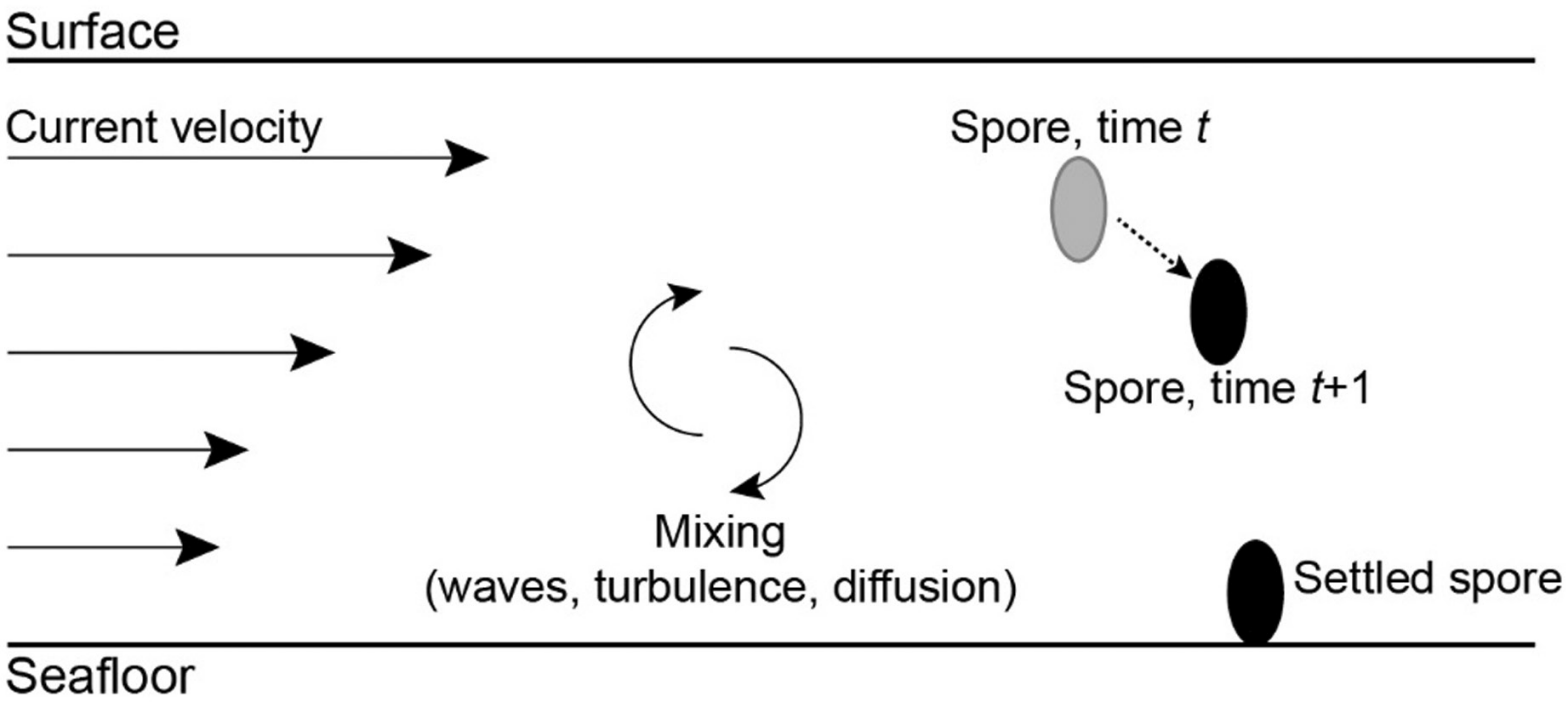
Schematic of the spore dispersal model. Simulated spores are released from a specific height in the water column. Horizontal and vertical movement in the water column depends on the current velocity, which changes with depth, and mixing. Once spores reach the seafloor, they are assumed to settle in place and no additional movement is allowed.

The numerical model used for spore dispersal estimates the time and horizontal distance potentially traveled by spores, from the time they are released at a prescribed height in the water column to the moment they settle on the seafloor. It is conceptual in character, quantifying the linear distances over which spores can be expected to be carried under particular, fixed oceanographic conditions. In this regard, the construct we employ contrasts with complicated “tactical” models that strive to predict convoluted trajectories spores might take in specific locations at specific times, and in situations when hydrographic conditions actively change. The core elements of the model, including the underlying mathematical equations and computational solution approaches, have been described elsewhere (Gaylord et al., 2002, 2004, 2006). We limit the description here to a qualitative narrative (Figure 1). Two‐dimensional movements of spores, in the horizontal and vertical directions, are driven by current velocities, interactions of currents with waves, and turbulence‐induced mixing arising from bottom friction. Although spore swimming and molecular diffusivity also contribute to spore movement, these processes are negligible beyond microscopic scales. Water velocity profiles were modeled across the water column and through time as functions of the water depth, current speed and direction relative to waves, wave period and height, seafloor roughness and boundary shear stress, and the kinematic viscosity of water. For each model simulation, spores were released at a specified height in the water column and were tracked through time. At each time point, spores moved horizontally following the horizontal water velocity at their respective depth, and they moved vertically according to their sinking speed and the local intensity and scales of mixing at their respective depths (Grant & Madsen, 1979, 1986; McNair et al., 1997; Wiberg & Smith, 1983). The vertical mixing processes (e.g., turbulent eddies move some water masses higher in the water column and some masses lower) were incorporated into the model as a depth‐dependent random walk (Gaylord et al., 2002, 2004, 2006; McNair et al., 1997). At the end of each time step, the new horizontal and vertical positions of every spore were updated, thereby informing the water velocity and mixing conditions of the following time step. Once the spores contacted the seafloor, they were assumed to settle. The model was allowed to run for a simulated duration that corresponds to the viability of spores in nature. Despite spores being released at the same starting point, the vertical gradients in horizontal velocity and the stochasticity associated with turbulent mixing resulted in a distribution of settling times and distances. The version of the numerical model implemented here was adjusted from the original (Gaylord et al., 2002) through the incorporation of *Nereocystis*‐specific spore traits and release heights and regionally appropriate current, wave, and turbulence conditions as described below.

### 2.2 Parameterizing the dispersal model

Oceanographic conditions in the model were tuned to represent the general flow conditions experienced by *Nereocystis* populations along the California coast. Current velocities and wave characteristics were obtained from local field measurements and regional trends (Elsmore et al., 2022, 2023; Gaylord et al., 2006). Model parameters are listed in Table 1. Relevant flow conditions were used rather than a detailed time series of observed flows because the aim was to understand how dispersal is shaped by the reproductive strategies of *Nereocystis* rather than to forecast dispersal from certain populations under specific flow regimes. Previous applications of this model approach for *Macrocystis* spore dispersal assessed the model's sensitivity to flow conditions and its ability to accurately predict the dispersal of spores from first principles (Gaylord et al., 2006).

**TABLE 1:** Tunable parameters are used in the dispersal model.

Parameter	Value	References
Spore viability (simulation duration)	3 d	Schiel and Foster (2006)
Water depth	10 m	Elsmore et al. (2022, 2023), Gaylord et al. (2006)
Significant wave height	0.5 m	Elsmore et al. (2022, 2023), Gaylord et al. (2006)
Wave period	12 s	Elsmore et al. (2022, 2023), Gaylord et al. (2006)
Current velocity and direction at 1.5 m above the seabed	0.05 m s^−1^	Elsmore et al. (2022, 2023), Gaylord et al. (2006)
Bottom roughness	0.8 cm	Gaylord et al. (2006)
Kinematic viscosity of seawater	1.0 × 10^−6^ m^2^ s^−1^	Vogel (1996)

*Note*: Waves were assumed to propagate shoreward, perpendicular to the alongshore current. Bottom roughness was selected to resemble the flat, sandy seafloor that typically separates patches of *Nereocystis* habitat.

One of the unique reproductive strategies of *Nereocystis* is a bimodal spore release height: spores can be released from the sori into the water column at the water surface while the sori are attached to the blades, and near the seafloor after the sori abscise and sink through the water column. To incorporate this trait in the model, cohorts of spores were released at the surface (10 m above the benthos), near the seafloor (5 cm above the benthos), or at both heights (bimodal spore release; half of the spore cohort released at each height). The bimodal release used here was intended to be an average between the single‐point releases that could occur at either the surface or near the seafloor.

Sinking speeds of *Nereocystis* spores were measured experimentally following the methodology of Gaylord et al. (2002). Two replicate trials were conducted on different dates (October 2021 and July 2022), each using a unique starting concentration of suspended spores (4.35 × 10^6^ spores mL^−1^; 2.38 × 10^5^ spores mL^−1^, respectively). The spores were obtained from fertile sporophylls collected from several individuals in different locations at Russian Gulch, California (38°28′ N, 123°9′ W). Sporophylls were transferred in coolers with seawater to the University of California, Davis' Bodega Marine Laboratory (Bodega Bay, CA). There, the sporophylls were rinsed with a 1% iodine solution for 30 s, then rinsed immediately with deionized water for 30 s and filtered seawater (1 μm) for 1 min. Then, blades were wiped gently and layered between moist paper towels and placed in a dark temperature‐controlled room (10°C) for 12 h. Spores were released by re‐immersing all the sporophylls in a beaker with filtered seawater (1 μm) at 15°C in ambient room light and stirring occasionally over the course of 1 h. Sporophylls were removed, the solution was filtered through a 40 μm mesh to further strain out particles and break up mucus clumps, and the trials ensued directly after. The spore suspension solution was stirred to evenly distribute spores and used to fill nine beakers to a depth of 40 mm (80 mL), each containing a glass coverslip previously centered on the bottom of the beaker. For the duration of a trial, beakers were placed in a constant‐temperature room at 10°C in the dark to minimize convective fluid motions. Every hour for 9 h, one cover slip was carefully retrieved and placed on top of the grid of a hemocytometer to determine the density of settled spores. A total of 10 sectors (0.2 mm × 0.2 mm) from the hemocytometer grid were counted per time point (Bros & Cowell, 1987). Spores adhere firmly to glass upon settling (Reed et al., 1991), thus dislodgement and loss of spores during retrieval were assumed negligible. Spore settlement density increased steadily until leveling off; this latter condition corresponded to when most spores had settled out of the water column and was used in conjunction with water depth (40 mm) to calculate the sinking speed (sinking speed = depth divided by leveling‐off time). This calculation is based on the assumption that the number of settled spores on a cover slip increases over time as long as some spores remain in suspension, and that settlement stops once all spores have sunk to the bottom.

Spore viability (i.e., the ability to develop into a gametophyte after settling), and thus the maximum dispersal time allowed in our model, was set to 3 days, which is a modestly conservative estimate. The spores of many kelps and other macroalgae settle out of the water column in several hours but have also been reported to be viable for up to 5 days (Edwards, 2022; Fletcher & Callow, 1992; Santelices, 1990; Schiel & Foster, 2006).

### 2.3 Implementing the dispersal model

In each simulation of the model, a cohort of 1000 virtual spores was released at heights corresponding to the surface, seafloor, and bimodal release heights observed in *Nereocystis*, while holding oceanographic conditions constant. This cohort size is orders of magnitude smaller than the number of spores actually released by an adult kelp per day in nature but is sufficient for our purposes, as we are interested exclusively in quantifying the shape of a resulting dispersal distribution. We also included a simulation with release heights following a random uniform distribution (across all depths) to serve as a null model. Only the dispersal distances of spores that settled while viable (<3 days in the modeled time) were considered in our subsequent analyses. After each simulation, we calculated the dispersal exceedance probability, following Gaylord et al. (2002), and in separate analyses, compared the frequencies of spores that traveled different dispersal ranges corresponding to short‐, intermediate‐, and long‐distance dispersal.

We conducted local sensitivity analyses of the percent of settled spores for the spore release height parameter, the ratio of spore release at the surface versus the seafloor in bimodal spore release, and spore sinking speed. Our investigations of *Nereocystis* were followed with a model simulation that employed the known spore release height (42 cm) and spore sinking speed (0.0012 mm s^−1^) of *Macrocystis* (Gaylord et al., 2002, 2006) to understand how the two dominant canopy‐forming kelp species differ in their dispersal potential when all other conditions were held constant.

## 3 RESULTS

### 3.1 Spore sinking speed

**FIGURE 2:**
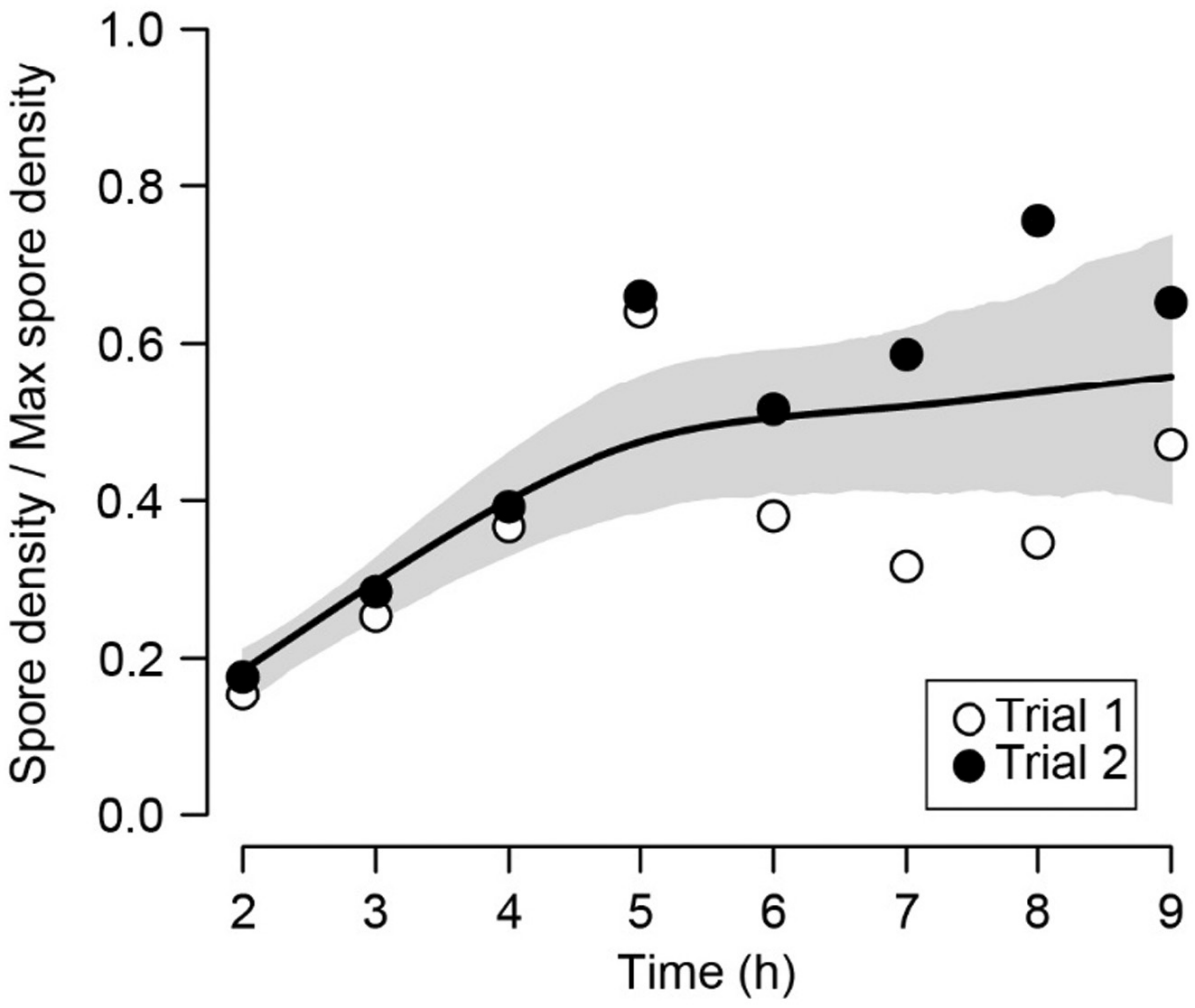
Spore settlement densities over time from an experimental assay to determine sinking speeds of *Nereocystis* spores. Data are normalized to the maximum settlement density observed in each trial (open circles = trial 1, closed circles = trial 2). Variation in densities after 5 h was likely due to seasonal differences in spores, such as viability. The black line shows a cubic splining curve calculated via the “smooth. spline” function in R Statistical Software (https://www.r‐project.org), and the shaded area represents 95% confidence intervals.

The density of *Nereocystis* spores settling on glass coverslips leveled off after approximately 5 h (Figure 2), indicating a mean sinking speed of 0.0022 mm s^−1^. This value falls within the range of sinking speeds observed for the spores and propagules of other macroalgae. In particular, it is 83% faster than *Macrocystis* spores (Fletcher & Callow, 1992; Gaylord et al., 2002; Norton & Fetter, 1981).

### 3.2 Dispersal distributions

**FIGURE 3:**
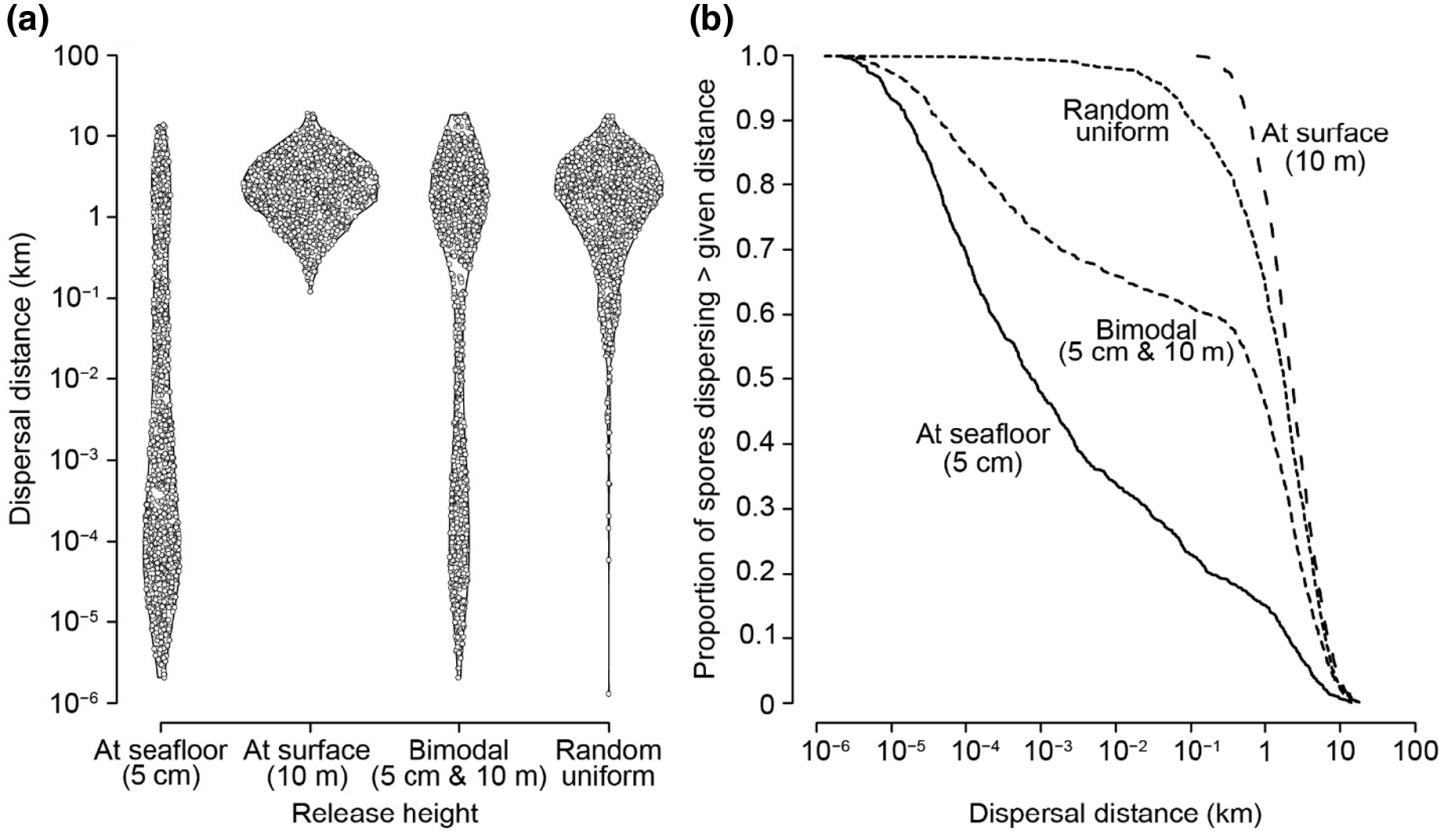
Predicted dispersal distances of virtual spores released at different heights in the water column. (a) Dispersed distances of spores in each simulation (*n* = 1000 spores) are shown as violin plots where the width of the plot indicates the relative frequency of spores settling at that distance. Points show individual spores and are arranged to convey the relative frequency of settlement at each distance. Note that the vertical axis is on a logarithmic scale and that distances <10^−4^ km are shorter than 10 cm. (b) Proportion of spores dispersing beyond a given distance. Each curve is labeled according to its respective spore release height.

The dispersal distributions of model spores released near the seafloor, at the water surface, at both (bimodal), or randomly (null model) are shown in Figure 3. Most spores released near the seafloor were predicted to settle within 1 m of their origin (median distance = 75 cm), although some spores dispersed >10 km, which is in agreement with field observations by Reed et al. (1988) for *Macrocystis*. Spores released at the surface dispersed between 100 m and 20 km (median = 2.2 km). Bimodal spore release, with 500 spores released at 5 cm and 500 spores at 10 m, yielded a broad range of dispersal distances: a cluster of spores settled within 1 m of their origin while another cluster dispersed between 1 and 10 km (median = 779 m). Modeled spores released at random heights in the water column tended to disperse long distances (median = 1.8 km) with relatively few spores settling within 10 m of their origin.

### 3.3 Sensitivity analyses

**FIGURE 4:**
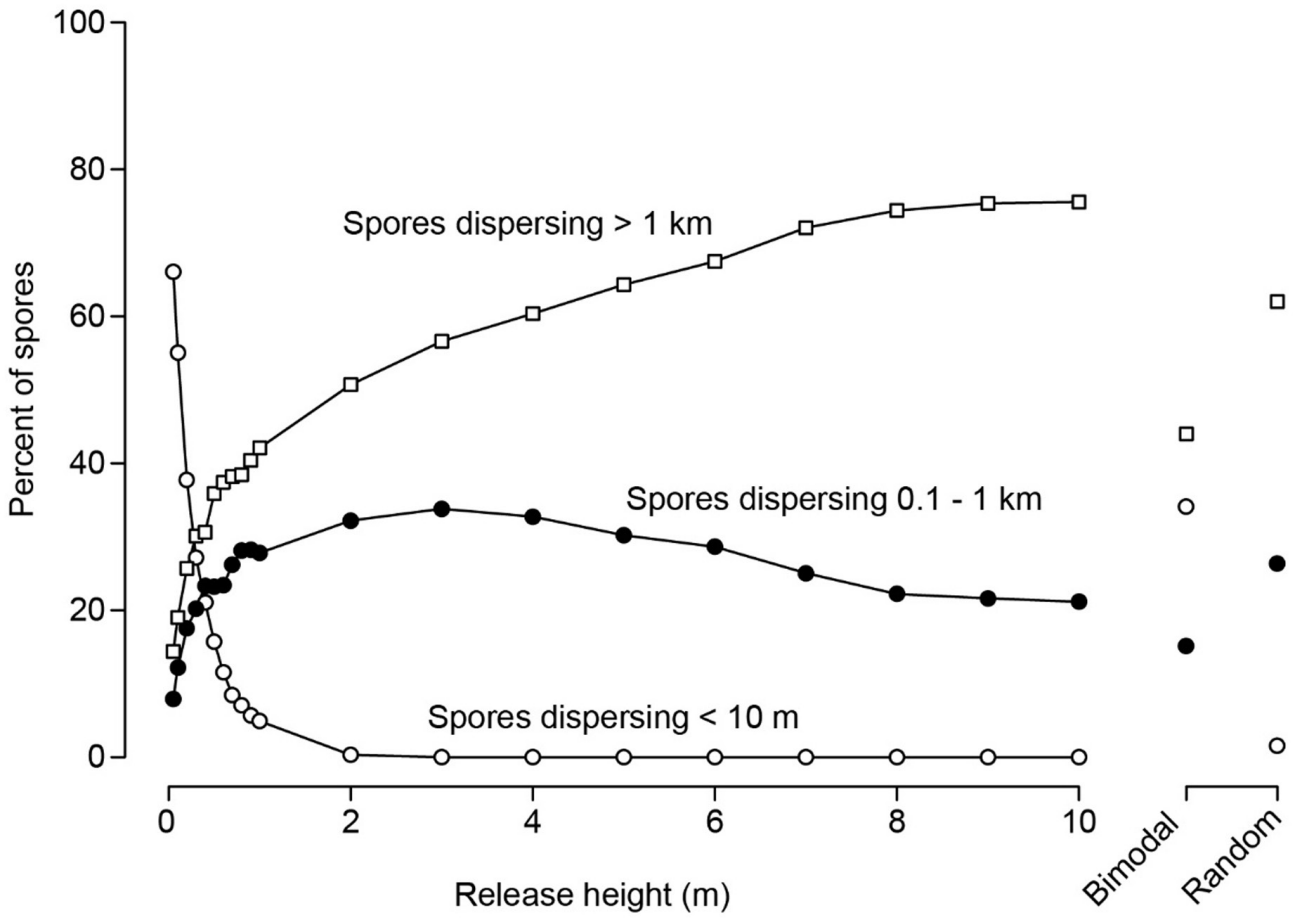
Results of sensitivity analyses for spore release height. The percent of spores dispersing short (<10 m), intermediate (0.1–1 km), and long distances (>1 km) are shown for localized release heights ranging from 5 cm to 10 m. Results for bimodal release heights and a random uniform distribution of heights are shown at right.

**FIGURE 5:**
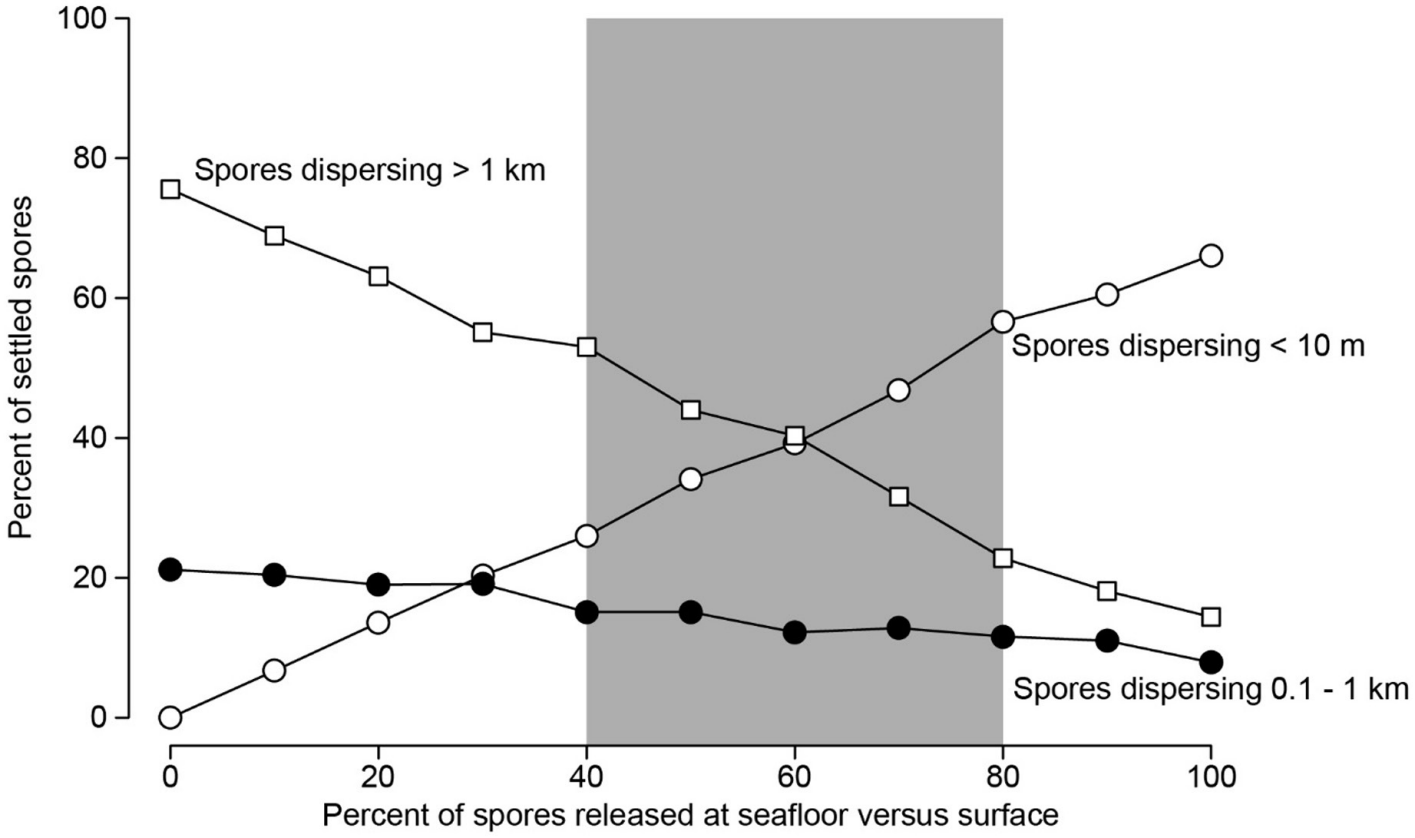
Sensitivity analysis of seafloor versus surface release contributions to bimodal spore release. The percent of spores dispersing short (<10 m), intermediate (0.1–1 km), and long distances (> 1 km) are shown for spores released only at the surface (0%), only near the seafloor (100%), and combinations of the two. The bimodal spore release used in other analyses here represents a 50% seafloor, 50% surface release. Substantial dispersal to both short and long distances (i.e., >20% of spores settling in each region) is possible when 40%–80% of spores are released near the seafloor and the remainder are released at the surface (shaded region).

Dispersal distances were most sensitive to changes in release height when release heights were <2 m above the seafloor (Figure 4). When simulated release occurred at 5 cm from the seafloor, most spores (66%) settled within 10 m of their origin, but very few (only 5%) settled within this distance when released at 1 m from the seafloor. Correspondingly, an increasing proportion of spores settled farther from their origin as release height increased. With bimodal release heights, 34% of spores settled within 10 m of their origin (“short‐distance dispersal”), and nearly 60% dispersed intermediate (0.1–1 km) and long (>1 km) distances. Dispersal distributions with bimodal release heights were similar to distributions resulting from a single point release at approximately 50 cm, although small variations in point release heights around 50 cm might yield substantially different dispersal distributions. Transport of at least 20% of spores into each of the short‐ and long‐distance regions was possible with bimodal release heights when 40–80% of spores were released at the seafloor and the remainder were released at the surface (Figure 5). In contrast, random release heights mostly yielded intermediate (26%) and long‐distance (62%) dispersal with infrequent, short‐distance dispersal (<2%). Random release heights had a similar dispersal distribution as the point release at 5 m, which is expected given that the mean release height in the random release simulation should be close to 5 m.

**FIGURE 6:**
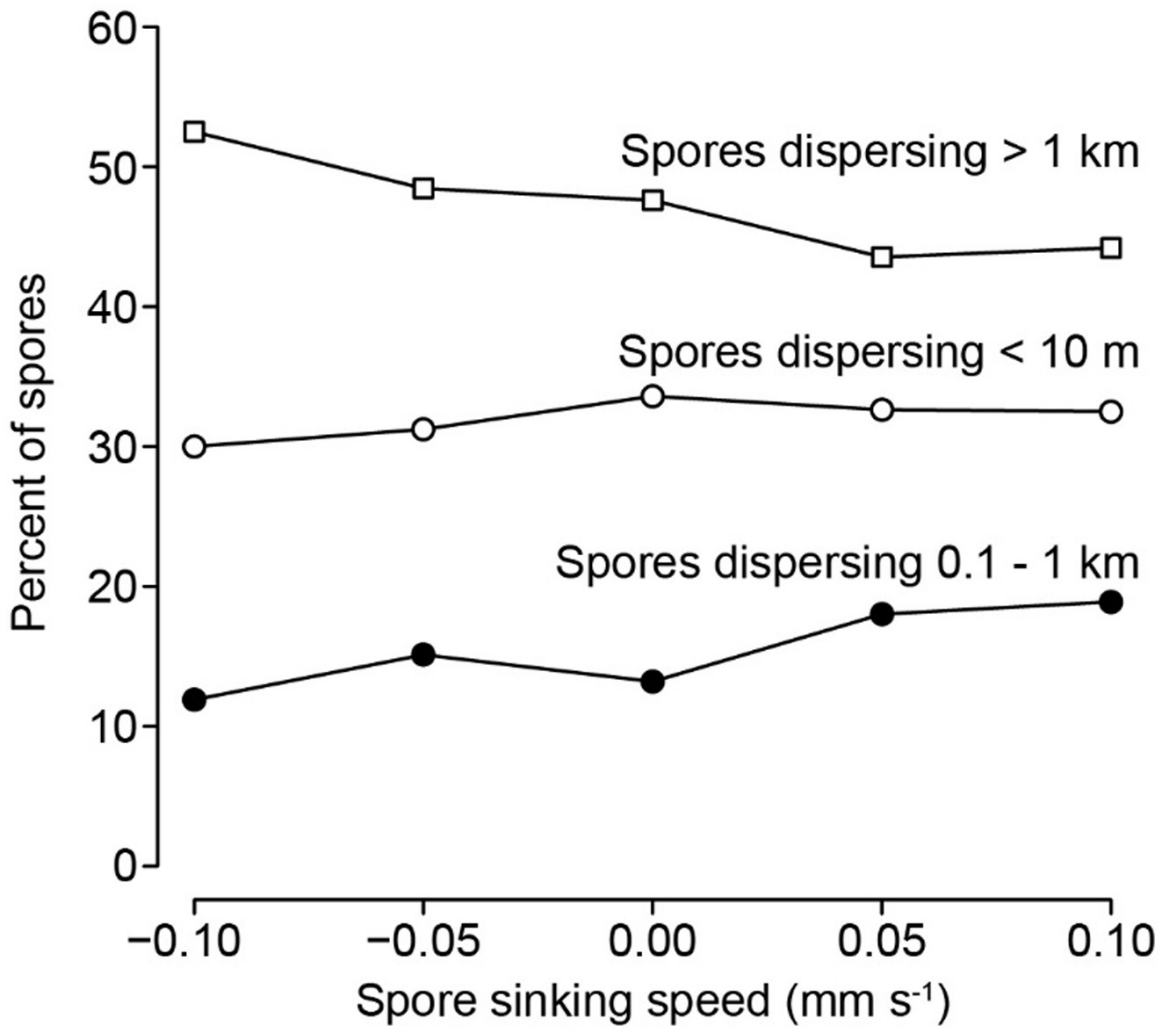
Percent of spores dispersing different distance intervals, plotted for a series of simulations with spore sinking speeds ranging from negative values (swimming upward, away from the seafloor) to positive values (swimming or sinking downward, toward the seafloor). All simulated spores shown here were released from bimodal release heights.

Spore sinking speed had little influence on dispersal distributions, even if spores were neutrally buoyant or actively swimming upward away from the seafloor (Figure 6). Although *Nereocystis* spores are not believed to swim upward (e.g., as a positive phototaxis), upward swimming behavior was included in the sensitivity analysis because such behavior has been observed in the spores of other seaweeds (e.g., the brown alga *Ectocarpus siliculosus*; Amsler et al., 1999). Regardless, the small effect of sinking speed on dispersal distributions is not surprising given that sinking speeds are much slower than the velocities associated with turbulent mixing (Gaylord et al., 2002, 2004, 2006).

**FIGURE 7:**
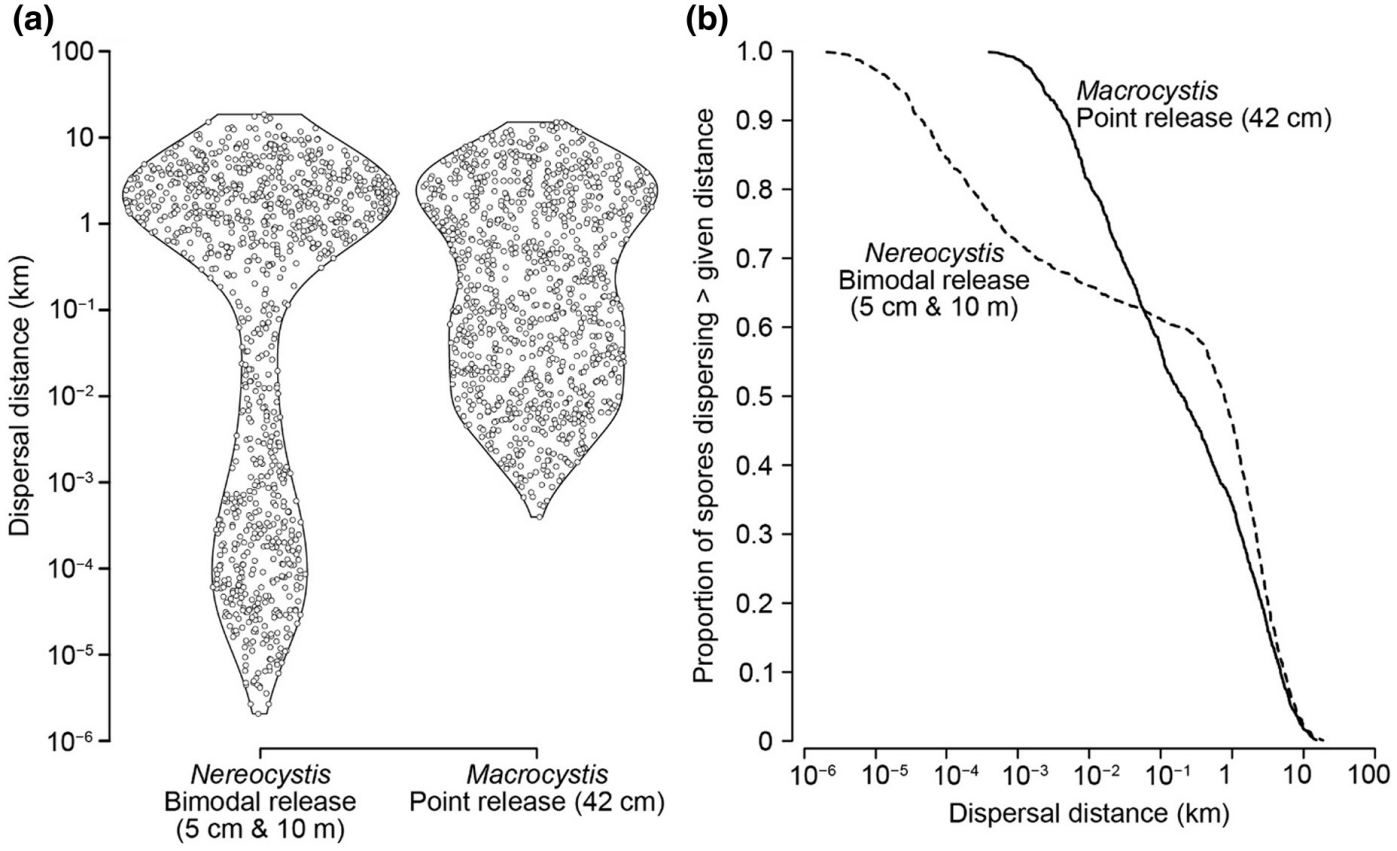
Comparisons of spore dispersal for *Nereocystis* and *Macrocystis* under the same flow conditions. (a) Violin plots showing distributions of dispersal distances, with points representing individual spores (*n* = 1000 spores per simulation). (b) Proportion of spores dispersing beyond a given distance for *Nereocystis* (dashed line) and *Macrocystis* (solid line).

*Nereocystis* had nearly twice the amount of spores settling within 10 m (34%) compared to *Macrocystis* (18%) (Figure 7). In contrast, both species had similar amounts of intermediate‐distance dispersal (0.1–1 km; *Nereocystis* = 15%, *Macrocystis* = 23%) and long‐distance dispersal (>1 km; *Nereocystis* = 46%, *Macrocystis* = 34%).

## 4 DISCUSSION

Our numerical model of spore dispersal, developed from first principles, reveals how a key anatomical trait (namely, the abscission of sori from the blades of *Nereocystis*) and the processes surrounding it, can lead to vastly different spore release patterns as compared to other canopy‐forming kelp, such as *Macrocystis* (Gaylord et al., 2002). In particular, *Nereocystis* can exhibit strong short‐ and long‐distance spore dispersal, each facilitated by this species' dual configuration of spore release. In our simulations with bimodal release heights, where half the spores were released from the water's surface and half from the near the seafloor, 34% of spores dispersed short distances (<10 m) and just over 40% dispersed long distances (>1 km). The equal numbers of spores released at the surface and seafloor were intended as an average between the two extremes of single‐point releases (i.e., all spores released at either the surface or seafloor). However, our sensitivity analyses suggest that bimodal dispersal distributions are robust to moderate variation in the percentage of seafloor versus surface releases. That is, when 40%–80% of spores are released near the seafloor and the remainder is released at the surface, short‐ and long‐distance regions receive more than 20% of spores each (Figure 5). The exact ratio of seafloor versus surface spore release exhibited by *Nereocystis*, as well as plasticity in the ratio, remain unclear. Below we discuss how spore dispersal in *Nereocystis* is shaped by its anatomy and what this means for the resilience and connectivity of kelp populations, as well as how conservation efforts can capitalize on spore dispersal distributions to tailor restoration of collapsed *Nereocystis* forests.

### 4.1 Local retention and connectivity

Nearly one‐third of modeled spores settled within 10 m of their release point. Short‐distance dispersal complements the annual life history of *Nereocystis* because it permits successive generations of kelp to recruit near areas with viable substrata where prior cohorts have succeeded (Gaylord et al., 2006). Another benefit of short‐distance dispersal is that it facilitates spores settling at high enough densities to allow gametes released by subsequent gametophyte stages to fertilize (Dayton, 1985; Reed et al., 1997), although there can also be costs of self‐fertilization if the microscopic stages originate from the same adult (Raimondi et al., 2004). Short‐distance dispersal can additionally enhance the self‐replenishment of forests, permitting kelp populations to increase in density or area. This feature can decrease the population's probability of collapse or susceptibility to catastrophic disturbances such as storms or anomalous temperatures (Rogers‐Bennett & Catton, 2019; Seymour et al., 1989). Elevated sporophyte abundance or density resulting from high self‐replenishment can strengthen the influence of the kelp on local biodiversity and hydrographic processes (Elsmore et al., 2022, 2023; Gaylord et al., 2006, 2012). However, if only a few individuals contribute to self‐replenishment and sexual reproduction in a kelp forest, a bottleneck for genetic variation can arise that eventually makes the population vulnerable to heat waves and other environmental stressors (Alsuwaiyan et al., 2021; Johnson & Gaines, 1990; Ronce, 2007; Wernberg et al., 2018).

Long‐distance dispersal of spores can add individuals to nearby kelp forests that are struggling or reestablish populations that have disappeared (the “rescue effect”; Brown & Kodric‐Brown, 1977). Localized, temporary loss of forests is a well‐known feature of canopy‐forming kelps (Schiel & Foster, 2015). The fact that individual kelp forests commonly undergo strong declines or even localized extinctions means that the persistence of the overall metapopulation depends on recolonization processes that foster recovery of decimated stands (Reed et al., 2006). Long‐distance dispersal can also increase genetic variation in kelp populations, improving resiliency to environmental challenges, and minimizing negative consequences of inbreeding that can arise if kelps undergo self‐fertilization (Johnson & Gaines, 1990; Raimondi et al., 2004; Ronce, 2007).

### 4.2 Sori abscission facilitates bimodal spore release heights

The unique spore dispersal distributions of *Nereocystis* occur because spores can be released while the sori are attached to kelp blades at the water surface or after the sori abscise from the blades and sink to the seafloor. Sorus abscission, and therefore bimodal spore release, is reported in only one other seaweed species, the kelp *Lessoniopsis littoralis* (MacMillan, 1900; Walker, 1980). However, *L. littoralis* has a relatively short thallus (2 m) and develops reproductive tissues near its holdfast (Abbott & Hollenberg, 1976); it also is primarily an intertidal species subject to surf‐zone flows as opposed to the coastal currents most relevant to *Nereocystis*. Therefore, *L. littoralis* likely does not achieve a bimodal spore release and its spore dispersal distribution likely does not reach the same relative proportions of short‐ and long‐distance dispersal as *Nereocystis* (Figure 4). Beyond the location of sorus development, there is uncertainty around the relative frequency of spore release before versus after sorus abscission, and what environmental factors might influence this frequency (Amsler & Neushul, 1989). The symmetric spore release distribution employed in the dispersal model here (50% of spores released at the water surface, 50% near seafloor) is intended as an average estimate because spore release could be induced earlier or later, relative to sorus abscission, based on local environmental conditions. Bimodal releases that deviate slightly from a symmetric release distribution (i.e., 40%–80% of spores released near the seafloor) will still deliver at least one‐fifth of spores across short and long distances, each. However, significant deviations from a 50% seafloor, 50% surface bimodal release will yield dispersal distributions that resemble point releases at either the surface or seafloor (Figure 5). Distinct spore release patterns of individual sori can also be immaterial because an individual *Nereocystis* produces multiple sori across multiple blades. Thus, the partitioning of spores across release heights can vary among sori, among blades, among individuals, and among populations. Further investigation is needed to quantify spore release patterns from sori and how they vary across levels of organization in the kelp.

### 4.3 Bet‐hedging and plasticity reduce stochasticity in spore dispersal

Spore release traits that foster simultaneous short‐ and long‐distance dispersal resemble a form of bet‐hedging (Gadgil, 1971). Local dispersal facilitates fitness by fostering successful recruitment in habitat where spore settlement is high and reproduction likely, with the caveat that inbreeding depression or intraspecific competition could at times degrade survival. Distant dispersal delivers spores to other sites whose environmental conditions might be decoupled from factors driving mortality within the originating forest. However, spores transported to distant sites could experience “wastage” if densities are too low to enable fertilization by subsequent life stages. The latter scenario is less likely for spores delivered to sites with extant forests where an additional source of spores is available. Notably, any bet‐hedging of this type is achieved largely through the anatomical positioning of the sori rather than the behavior of spores – that is, kelp spores swim negligibly slow relative to the flow velocity of local turbulence and mixing (Gaylord et al., 2002), and variation in spore swimming speeds has little effect on spore dispersal distributions (Figure 6).

Some of the stochasticity involved with dispersal could be reduced if spore release and sori abscission are initiated or influenced by environmental conditions that facilitate short‐ and long‐distance dispersal. For instance, *Nereocystis* populations in central California exhibit a strong diel periodicity in sori abscission, with abscission occurring primarily at dawn, presumably to maximize the photosynthetic potential of spores (Amsler & Neushul, 1989), whereas sori abscission in populations near British Columbia is aligned with the anomalously strong tidal currents that occur in the region (Walker, 1980). To the extent that spore release by *Nereocystis* is responsive to the environment, the kelp can be capable of locally tuned allocations to short‐ versus long‐distance spore dispersal that could influence the persistence and connectivity of *Nereocystis* populations across a range of latitudes and hydrodynamic conditions (Johnson & Koehl, 1994; Reed et al., 2006).

Additional forms of plasticity help reduce the stochasticity of dispersal in *Nereocystis*. First, the kelp can remain in its microscopic gametophyte life stage for an extended period of time, similar in concept to seed banks that are used with terrestrial plants (Edwards, 2000, 2022; Friedman, 2020; Gremer et al., 2012; Reed et al., 2006; Schiel & Foster, 2006). As a result, individuals can persist as gametophytes until environmental conditions are suitable for advancing to the next life stage. Remaining dormant can also aid recruitment on sparsely available substrata or elevate genetic variation within a population, as gametophytes from multiple populations can accumulate in each location before collectively moving on to sexual reproduction and development into sporophytes.

Another life history trait that contributes to long‐distance dispersal is the release of spores by drift kelp. Developmental plasticity of the microscopic life stages of *Nereocystis* allows spores to disperse long distances via rafting and then remain dormant until environmental conditions become favorable. Large rafts of drift *Nereocystis* can travel substantial distances while releasing spores, thereby enhancing the kelp's long‐distance dispersal (Hobday, 2000; Kidder, 2006; Koehl & Wainwright, 1977; Thiel, 2003). In other kelp, including *Macrocystis*, individual sporophytes release spores while adrift, although they can experience herbivory and general deterioration that reduces sporophyte size and consequently reduces the sporophylls available to release spores (Hernández‐Carmona et al., 2006). Drift kelp can also be quickly washed ashore or be exported offshore, limiting the temporal window in which spores can be released near suitable habitats (Harrold & Lisin, 1989; Hobday, 2000; Kingsford, 1995; ZoBell, 1971). The small number of sporophytes in a drift raft and the long distances traveled – albeit briefly before washing ashore or offshore – suggests that spore release by drift kelp is a mechanism for occasional long‐distance dispersal, benefiting the kelp by biogeographic expansion or by increasing genetic variation in distant populations (Reed et al., 2006). It also can be a mechanism by which algae colonize oceanic islands (van den Hoek, 1987). Cumulatively, *Nereocystis* possesses several complementary strategies to ensure both short‐ and long‐distance dispersal, yet the maximum extent to which these traits increase the persistence or recolonization of *Nereocystis* populations remains unknown.

### 4.4 Species‐specific conservation efforts

Recent large‐scale declines of *Nereocystis* populations have signaled a need for the conservation and restoration of the kelp (Krumhansl et al., 2016; Rogers‐Bennett & Catton, 2019). However, it is unclear how conservation and restoration strategies that are established for other kelp species can be tailored to *Nereocystis* (Eger et al., 2022). In particular, there is a long history and wealth of data resulting from the restoration of *Macrocystis* in California (reviewed by Eger et al., 2022), raising the question of how and whether the unique life history of *Nereocystis* warrants different management approaches in the same region. Expanding on interspecific differences outlined in the Introduction above, the numerical dispersal model used here reveals that *Nereocystis* has a larger capacity for short‐distance dispersal (<10 m) than *Macrocystis*, but the similar capacity for long‐distance dispersal (>1 km) (Figure 7). Our model compares the dispersal of the two kelps under equivalent flow conditions and depths, although the two species can in fact experience separate conditions in their respective habitats. Variation in oceanographic conditions, which have been characterized in previous studies (Gaylord et al., 2002, 2004, 2006), can augment the dispersal distributions reported here. For instance, increased current velocities and water depths can extend the length scales of dispersal, whereas reduced current velocities and water depths can constrict dispersal. Under the standardized model conditions used here, we can conclude that self‐replenishment via local retention can be more important for *Nereocystis* than for *Macrocystis*, which can be correlated with life histories – that is, *Nereocystis* sporophytes are generally annuals and must replace themselves in the same location year after year for a population to persist, whereas *Macrocystis* sporophytes are perennials and their populations can persist without annual replenishment by spores (Reed et al., 2006).

Similarities in long‐distance spore dispersal between *Nereocystis* and *Macrocystis* suggest that population connectivity, though it can be stochastic, is an essential process in maintaining metapopulation viability and sufficient genetic variation in these species (Reed et al., 2006). Connectivity can avoid the deleterious effects of inbreeding (Raimondi et al., 2004) and enable populations to withstand environmental challenges such as thermal stress and disease (Alsuwaiyan et al., 2021; Wernberg et al., 2018). Past work with *Macrocystis* showed that the likelihood of spores settling in distant locations increases with proximity to a pre‐existing population (Reed et al., 2006), and the same is likely true for *Nereocystis*. Re‐establishment of isolated, extinct kelp patches is also likely to be facilitated by nearby kelp populations of large size, which have greater overall spore production. Local reserves of microscopic gametophytes that can remain on the seafloor where a kelp patch once grew can also bolster recolonization processes (Reed et al., 2006).

Effectively adapting management strategies from *Macrocytis* to *Nereocystis* relies on consideration of *Nereocystis'* short‐distance dispersal and annual life cycle. In an interconnected metapopulation of multiple kelp forests, we expect that the supply of kelp spores to a particular location will increase with the size and proximity of a source forest (Reed et al., 2006). Thus, achieving kelp spread starts with establishing viable, sustained sources (i.e., kelp patches) that are placed strategically near other potential reefs or struggling kelp forests and that have the potential to increase in size. For *Nereocystis*, facilitating growth in forest size requires adequate local retention of spores, which can be achieved through transplanting reproductive sporophytes or sori to a focal reef (Eger et al., 2022), or decreasing densities of voracious consumers of kelp after outbreaks (e.g., purple urchin *Strongylocentrotus purpuratus*) to improve survivorship of reproductive adults (Miller et al., 2022). In contrast, analogous outcomes for *Macrocystis* can be more effectively achieved by long‐term investment in individual sporophytes, which can survive and produce spores for multiple years (Schiel & Foster, 2015).

Another major difference between giant kelp and bull kelp is the timing of spore release. Within their annual life cycle, *Nereocystis* sporophytes produce sori only after growing to maturity, so spore production is limited to a narrow seasonal window (Amsler & Neushul, 1989; Walker, 1980); *Macrocystis* sporophytes produce spores across multiple seasons with little apparent seasonal peak (Reed et al., 1997). Consequently, restoration of *Nereocystis* kelp forests could benefit most from seasonal management efforts that span early spring (during recruitment) through the late summer when spores are released, versus year‐round management of the perennial *Macrocystis* forests. *Nereocystis* management goals can leverage general approaches to kelp forest restoration, such as grazer suppression and out‐planting of stress‐tolerant kelp lines (Eger et al., 2022; Assis et al., 2023). However, efforts that are tightly focused on only a few key *Nereocystis* forests, which can then act as sources for other stands, could then promote the long‐term health and stability of a kelp metapopulation.

## AUTHOR CONTRIBUTIONS

**Nicholas P. Burnett:** Conceptualization (equal); formal analysis (equal); investigation (equal); software (equal); visualization (equal); writing – original draft (equal). **Aurora M. Ricart:** Funding acquisition (equal); investigation (equal); methodology (equal); validation (equal); writing – review and editing (equal). **Tallulah Winquist:** Investigation (equal); writing – review and editing (equal). **Alisha M. Saley:** Investigation (equal); methodology (equal); writing – review and editing (equal). **Matthew S. Edwards:** Funding acquisition (equal); writing – review and editing (equal). **Brent Hughes:** Funding acquisition (equal); writing – review and editing (equal). **Jason Hodin:** Funding acquisition (equal); writing – review and editing (equal). **Marissa L. Baskett:** Funding acquisition (equal); writing – review and editing (equal). **Brian Gaylord:** Conceptualization (equal); funding acquisition (equal); investigation (equal); methodology (equal); project administration (equal); resources (equal); software (equal); supervision (equal); validation (equal); visualization (equal); writing – review and editing (equal).

## FUNDING INFORMATION

This work was supported by a California Sea Grant Kelp Recovery Research Program Award (Project R/HCE‐15) to B. Gaylord, M. Baskett, A. Ricart, M. Edwards, M. Zippay, B. Hughes, S. Place, and J. Hodin.

## Supporting information


Appendix S1

## Data Availability

Data are available in the Supporting Information.

## References

[ece370177-bib-0001] Abbott, I. A. , & Hollenberg, G. J. (1976). Marine algae of California. Stanford University Press.

[ece370177-bib-0002] Abelson, A. , & Denny, M. (1997). Settlement of marine organisms in flow. Annual Review of Ecology and Systematics, 28, 317–339.

[ece370177-bib-0003] Allee, W. C. (1931). Animal aggregations, a study in general sociology. The University of Chicago Press.

[ece370177-bib-0004] Alsuwaiyan, N. , Vranken, S. , Filbee‐Dexter, K. , Cambridge, M. , Coleman, M. , & Wernberg, T. (2021). Genotypic variation in response to extreme events may facilitate kelp adaptation under future climates. Marine Ecology Progress Series, 672, 111–121.

[ece370177-bib-0005] Amsler, C. D. , & Neushul, M. (1989). Diel periodicity of spore release from the kelp *Nereocystis luetkeana* (Mertens) Postels et Ruprecht. Journal of Experimental Marine Biology and Ecology, 134, 117–127.

[ece370177-bib-0006] Amsler, C. D. , Shelton, K. L. , Britton, C. J. , Spencer, N. Y. , & Greer, S. P. (1999). Nutrients do not influence swimming behavior or settlement rates of *Ectocarpus siliculosus* (Phaeophyceae) spores. Journal of Phycology, 35, 239–244.

[ece370177-bib-0007] Assis, J. , Alberto, F. , Macaya, E. C. , Castilho Coelho, N. , Faugeron, S. , Pearson, G. A. , Ladah, L. , Reed, D. C. , Raimondi, P. , Mansilla, A. , Brickle, P. , Zuccarello, G. C. , & Serrão, E. A. (2023). Past climate‐driven range shifts structuring intraspecific biodiversity levels of the giant kelp (*Macrocystis pyrifera*) at global scales. Scientific Reports, 13, 12046. 37491385 10.1038/s41598-023-38944-7 PMC10368654

[ece370177-bib-0008] Bernhardt, J. R. , & Leslie, H. M. (2013). Resilience to climate change in coastal marine ecosystems. Annual Review of Marine Science, 5, 371–392. 10.1146/annurev-marine-121211-172411 22809195

[ece370177-bib-0009] Breitbach, N. , Tillmann, S. , Schleuning, M. , Grünewald, C. , Laube, I. , Steffan‐Dewenter, I. , & Böhning‐Gaese, K. (2012). Influence of habitat complexity and landscape configuration on pollination and seed‐dispersal interactions of wild cherry trees. Oecologia, 168, 425–437. 21818655 10.1007/s00442-011-2090-1

[ece370177-bib-0010] Bros, W. E. , & Cowell, B. C. (1987). A technique for optimizing sample size (replication). Journal of Experimental Marine Biology and Ecology, 114, 63–71.

[ece370177-bib-0011] Brown, J. H. , & Kodric‐Brown, A. (1977). Turnover rates in insular biogeography: Effect of immigration on extinction. Ecology, 58, 445–449.

[ece370177-bib-0012] Burgess, S. C. , Baskett, M. L. , Grosberg, R. K. , Morgan, S. G. , & Strathmann, R. R. (2016). When is dispersal for dispersal? Unifying marine and terrestrial perspectives: When is dispersal for dispersal? Biological Reviews, 91, 867–882. 26118564 10.1111/brv.12198

[ece370177-bib-0013] Burnett, N. P. , & Koehl, M. A. R. (2018). Knots and tangles weaken kelp fronds while increasing drag forces and epifauna on the kelp. Journal of Experimental Marine Biology and Ecology, 508, 13–20.

[ece370177-bib-0014] Burnett, N. P. , & Koehl, M. A. R. (2020). Thallus pruning does not enhance survival or growth of a wave‐swept kelp. Marine Biology, 167, 52.

[ece370177-bib-0015] Carney, L. T. , Bohonak, A. J. , Edwards, M. S. , & Alberto, F. (2013). Genetic and experimental evidence for a mixed‐age, mixed‐origin bank of kelp microscopic stages in southern California. Ecology, 94, 1955–1965. 24279267 10.1890/13-0250.1

[ece370177-bib-0016] Carney, L. T. , & Edwards, M. S. (2006). Cryptic processes in the sea: A review of delayed development in the microscopic life stages of marine macroalgae. Algae, 21, 161–168.

[ece370177-bib-0017] Carney, L. T. , & Edwards, M. S. (2010). Role of nutrient fluctuations and delayed developmnet in gametophyte reproduction by *Macrocystis pyrifera* (Phaeophyceae) in Southern California. Journal of Phycology, 46, 987–996.

[ece370177-bib-0018] Carrano, M. W. , Carrano, C. J. , Edwards, M. S. , Al‐Adilah, H. , Fontana, Y. , Sayer, M. D. J. , Katsaros, C. , Raab, A. , Feldmann, J. , & Küpper, F. C. (2021). *Laminaria* kelps impact iodine speciation chemistry in coastal seawater. Estuarine, Coastal and Shelf Science, 262, 107531.

[ece370177-bib-0019] Carrano, M. W. , Yarimizu, K. , Gonzales, J. L. , Cruz‐López, R. , Edwards, M. S. , Tymon, T. M. , Küpper, F. C. , & Carrano, C. J. (2020). The influence of marine algae on iodine speciation in the coastal ocean. Algae, 35, 167–176.

[ece370177-bib-0020] Cavanaugh, K. , Siegel, D. , Reed, D. , & Dennison, P. (2011). Environmental controls of giant‐kelp biomass in the Santa Barbara Channel, California. Marine Ecology Progress Series, 429, 1–17.

[ece370177-bib-0021] Cie, D. K. , & Edwards, M. S. (2011). Vertical distribution of kelp zoospores. Phycologia, 50, 340–350.

[ece370177-bib-0022] Coleman, M. A. , Roughan, M. , Macdonald, H. S. , Connell, S. D. , Gillanders, B. M. , Kelaher, B. P. , & Steinberg, P. D. (2011). Variation in the strength of continental boundary currents determines continent‐wide connectivity in kelp: Boundary currents determine connectivity of kelp. Journal of Ecology, 99, 1026–1032.

[ece370177-bib-0023] Cowen, R. K. , & Sponaugle, S. (2009). Larval dispersal and marine population connectivity. Annual Review of Marine Science, 1, 443–466. 10.1146/annurev.marine.010908.163757 21141044

[ece370177-bib-0024] Dayton, P. K. (1985). Ecology of kelp communities. Annual Review of Ecology and Systematics, 16, 215–245.

[ece370177-bib-0025] Dayton, P. K. , Tegner, M. J. , Edwards, P. B. , & Riser, K. L. (1999). Temporal and spatial scales of kelp demography: The role of oceanographic climate. Ecological Monographs, 69, 219–250.

[ece370177-bib-0026] Edwards, M. , Konar, B. , Kim, J.‐H. , Gabara, S. , Sullaway, G. , McHugh, T. , Spector, M. , & Small, S. (2020). Marine deforestation leads to widespread loss of ecosystem function. PLoS One, 15, e0226173. 32130220 10.1371/journal.pone.0226173 PMC7055868

[ece370177-bib-0027] Edwards, M. S. (2000). The role of alternate life‐history stages of a marine macroalga: A seed bank analogue? Ecology, 81, 2404–2415.

[ece370177-bib-0028] Edwards, M. S. (2004). Estimating scale‐dependency in disturbance impacts: El Niños and giant kelp forests in the northeast Pacific. Oecologia, 138, 436–447. 14673640 10.1007/s00442-003-1452-8

[ece370177-bib-0029] Edwards, M. S. (2022). It's the little things: The role of microscopic life stages in maintaining kelp populations. Frontiers in Marine Science, 9, 871204.

[ece370177-bib-0030] Edwards, M. S. , & Estes, J. A. (2006). Catastrophe, recovery and range limitation in NE Pacific kelp forests: A large‐scale perspective. Marine Ecology Progress Series, 320, 79–87.

[ece370177-bib-0031] Edwards, M. S. , & Konar, B. (2012). A comparison of dragon kelp, *Eualaria fistulosa*, (Phaeophyceae) fecundity in urchin barrens and nearby kelp beds throughout the Aleutian Archipelago. Journal of Phycology, 48, 897–901. 27009000 10.1111/j.1529-8817.2012.01139.x

[ece370177-bib-0032] Eger, A. M. , Marzinelli, E. M. , Christie, H. , Fagerli, C. W. , Fujita, D. , Gonzalez, A. P. , Hong, S. W. , Kim, J. H. , Lee, L. C. , McHugh, T. A. , Nishihara, G. N. , Tatsumi, M. , Steinberg, P. D. , & Vergés, A. (2022). Global kelp forest restoration: Past lessons, present status, and future directions. Biological Reviews, 97, 1449–1475. 35255531 10.1111/brv.12850 PMC9543053

[ece370177-bib-0033] Elsmore, K. , Nickols, K. , Ford, T. , Cavanaugh, K. , Cavanaugh, K. , & Gaylord, B. (2022). *Macrocystis pyrifera* forest development shapes the physical environment through current velocity reduction. Marine Ecology Progress Series, 694, 45–59.

[ece370177-bib-0034] Elsmore, K. , Nickols, K. J. , Miller, L. P. , Ford, T. , Denny, M. W. , & Gaylord, B. (2023). Wave damping by giant kelp, *Macrocystis pyrifera* . Annals of Botany, mcad094, 133, 29–40. 10.1093/aob/mcad094 PMC11087658 37463436

[ece370177-bib-0035] Estes, J. A. , Tinker, M. T. , Williams, T. M. , & Doak, D. F. (1998). Killer whale predation on sea otters linking oceanic and nearshore ecosystems. Science, 282, 473–476. 9774274 10.1126/science.282.5388.473

[ece370177-bib-0036] Filbee‐Dexter, K. , Feehan, C. , & Scheibling, R. (2016). Large‐scale degradation of a kelp ecosystem in an ocean warming hotspot. Marine Ecology Progress Series, 543, 141–152.

[ece370177-bib-0037] Filbee‐Dexter, K. , & Scheibling, R. (2014). Sea urchin barrens as alternative stable states of collapsed kelp ecosystems. Marine Ecology Progress Series, 495, 1–25.

[ece370177-bib-0038] Fletcher, R. L. , & Callow, M. E. (1992). The settlement, attachment and establishment of marine algal spores. British Phycological Journal, 27, 303–329.

[ece370177-bib-0039] Fraser, C. I. , Dutoit, L. , Morrison, A. K. , Pardo, L. M. , Smith, S. D. A. , Pearman, W. S. , Parvizi, E. , Waters, J. , & Macaya, E. C. (2022). Southern Hemisphere coasts are biologically connected by frequent, long‐distance rafting events. Current Biology, 32, 3154–3160. 35679870 10.1016/j.cub.2022.05.035

[ece370177-bib-0040] Friedman, J. (2020). The evolution of annual and perennial plant life histories: Ecological correlates and genetic mechanisms. Annual Review of Ecology, Evolution, and Systematics, 51, 461–481.

[ece370177-bib-0041] Gadgil, M. (1971). Dispersal: Population consequences and evolution. Ecology, 52, 253–261.

[ece370177-bib-0042] Gascoigne, J. , & Lipcius, R. (2004). Allee effects in marine systems. Marine Ecology Progress Series, 269, 49–59.

[ece370177-bib-0043] Gaylord, B. , Nickols, K. J. , & Jurgens, L. (2012). Roles of transport and mixing processes in kelp forest ecology. Journal of Experimental Biology, 215, 997–1007. 22357593 10.1242/jeb.059824

[ece370177-bib-0044] Gaylord, B. , Reed, D. C. , Raimondi, P. T. , & Washburn, L. (2006). Macroalgal spore dispersal in coastal environments: Mechanistic insights revealed by theory and experiment. Ecological Monographs, 76, 481–502.

[ece370177-bib-0045] Gaylord, B. , Reed, D. C. , Raimondi, P. T. , Washburn, L. , & McLean, S. R. (2002). A physically based model of macroalgal spore dispersal in the wave and current‐dominated nearshore. Ecology, 83, 1239–1251.

[ece370177-bib-0046] Gaylord, B. , Reed, D. C. , Washburn, L. , & Raimondi, P. T. (2004). Physical–biological coupling in spore dispersal of kelp forest macroalgae. Journal of Marine Systems, 49, 19–39.

[ece370177-bib-0047] Gaylord, B. , Rosman, J. H. , Reed, D. C. , Koseff, J. R. , Fram, J. , MacIntyre, S. , Arkema, K. , McDonald, C. , Brzezinski, M. A. , Largier, J. L. , Monismith, S. G. , Raimondi, P. T. , & Mardian, B. (2007). Spatial patterns of flow and their modification within and around a giant kelp forest. Limnology and Oceanography, 52, 1838–1852.

[ece370177-bib-0048] Gierke, L. , Coelho, N. C. , Khangaonkar, T. , Mumford, T. , & Alberto, F. (2023). Range wide genetic differentiation in the bull kelp *Nereocystis luetkeana* with a seascape genetic focus on the Salish Sea. Frontiers in Marine Science, 10, 1275905.

[ece370177-bib-0049] Gonzales, J. , Tymon, T. , Küpper, F. C. , Edwards, M. S. , & Carrano, C. J. (2017). The potential role of kelp forests on iodine speciation in coastal seawater. PLoS One, 12, e0180755. 28800586 10.1371/journal.pone.0180755 PMC5553931

[ece370177-bib-0050] Graham, M. H. (2003). Coupling propagule output to supply at the edge and interior of a giant kelp forest. Ecology, 84, 1250–1264.

[ece370177-bib-0051] Graham, M. H. (2004). Effects of local deforestation on the diversity and structure of Southern California giant kelp forest food webs. Ecosystems, 7, 341–357.

[ece370177-bib-0052] Graham, M. H. , Vásquez, J. A. , & Buschmann, A. H. (2007). Global ecology of the giant kelp *Macrocystis*: From ecotypes to ecosystems. Oceanography and Marine Biology: An Annual Review, 45, 39–88.

[ece370177-bib-0053] Grant, W. D. , & Madsen, O. S. (1979). Combined wave and current interaction with a rough bottom. Journal of Geophysical Research: Oceans, 84, 1797–1808.

[ece370177-bib-0054] Grant, W. D. , & Madsen, O. S. (1986). The continental‐shelf bottom boundary layer. Annual Review of Fluid Mechanics, 18, 265–305.

[ece370177-bib-0055] Gremer, J. R. , Crone, E. E. , & Lesica, P. (2012). Are dormant plants hedging their bets? Demographic consequences of prolonged dormancy in variable environments. The American Naturalist, 179, 315–327. 10.1086/664459 22322220

[ece370177-bib-0056] Harrold, C. , & Lisin, S. (1989). Radio‐tracking rafts of giant kelp: Local production and regional transport. Journal of Experimental Marine Biology and Ecology, 130, 237–251.

[ece370177-bib-0057] Hernández‐Carmona, G. , Hughes, B. , & Graham, M. H. (2006). Reproductive longevity of drifting kelp *Macrocystis pyrifera* in Monterey Bay, USA. Journal of Phycology, 42, 1199–1207.

[ece370177-bib-0058] Hobday, A. (2000). Abundance and dispersal of drifting kelp *Macrocystis pyrifera* rafts in the Southern California Bight. Marine Ecology Progress Series, 195, 101–116. 10.1016/s0022-0981(00)00250-1 11018238

[ece370177-bib-0059] Hodin, J. , Ferner, M. C. , Heyland, A. , & Gaylord, B. (2018). Chapter 13: I feel that! Fluid dynamics and sensory aspects of larval settlement across scales. In T. J. Carrier , A. M. Reitzel , & A. Heyland (Eds.), Evolutionary ecology of marine invertebrate larvae (pp. 190–207). Oxford University Press.

[ece370177-bib-0060] Hoffmann, A. J. , & Santelices, B. (1991). Banks of algal microscopic forms: Hypotheses on their functioning and comparisons with seed bank. Marine Ecology Progress Series, 79, 185–194.

[ece370177-bib-0061] Hogan, J. D. , Thiessen, R. J. , Sale, P. F. , & Heath, D. D. (2012). Local retention, dispersal and fluctuating connectivity among populations of a coral reef fish. Oecologia, 168, 61–71. 21735201 10.1007/s00442-011-2058-1

[ece370177-bib-0062] Hondolero, D. , & Edwards, M. S. (2017). Changes in ecosystem engineers: The effects of kelp forest type on currents and benthic assemblages in Kachemak Bay, Alaska. Marine Biology, 164, 81.

[ece370177-bib-0063] Jackson, G. A. , & Winant, C. D. (1983). Effect of a kelp forest on coastal currents. Continental Shelf Research, 2, 75–80.

[ece370177-bib-0064] Jeon, B. H. , Yang, K. M. , & Kim, J. H. (2015). Changes in macroalgal assemblage with sea urchin density on the east coast of South Korea. Algae, 30, 139–146.

[ece370177-bib-0065] Johnson, A. , & Koehl, M. (1994). Maintenance of dynamic strain similarity and environmental stress factor in different flow habitats: Thallus allometry and material properties of a giant kelp. Journal of Experimental Biology, 195, 381. 9317997 10.1242/jeb.195.1.381

[ece370177-bib-0066] Johnson, M. L. , & Gaines, M. S. (1990). Evolution of dispersal: Theoretical models and empirical tests using birds and mammals. Annual Review of Ecology and Systematics, 21, 449–480.

[ece370177-bib-0067] Kidder, K. A. (2006). Ecology and life history of Nereocystis luetkeana in the South Slough estuary. University of Oregon.

[ece370177-bib-0068] Kingsford, M. J. (1995). Drift algae: A contribution to near‐shore habitat complexity in the pelagic environment and an attract for fish. Marine Ecology Progress Series, 116, 297–301.

[ece370177-bib-0069] Klinger, T. (1985). *Allocation of blade surface area to meiospore production in annual and perennial representatives of the genus* Laminaria. Masters thesis. University of British Columbia. 10.14288/1.0096141

[ece370177-bib-0070] Koehl, M. A. R. , & Wainwright, S. A. (1977). Mechanical adaptations of a giant kelp. Limnology and Oceanography, 22, 1067–1071.

[ece370177-bib-0071] Krumhansl, K. A. , Okamoto, D. K. , Rassweiler, A. , Novak, M. , Bolton, J. J. , Cavanaugh, K. C. , Connell, S. D. , Johnson, C. R. , Konar, B. , Ling, S. D. , Micheli, F. , Norderhaug, K. M. , Pérez‐Matus, A. , Sousa‐Pinto, I. , Reed, D. C. , Salomon, A. K. , Shears, N. T. , Wernberg, T. , Anderson, R. J. , … Byrnes, J. E. K. (2016). Global patterns of kelp forest change over the past half‐century. Proceedings of the National Academy of Sciences of the United States of America, 113, 13785–13790. 27849580 10.1073/pnas.1606102113 PMC5137772

[ece370177-bib-0072] Layton, C. , Vermont, H. , Beggs, H. , Brassington, G. B. , Burke, A. D. , Hepburn, L. , Holbrook, N. , Marshall‐Grey, W. , Mesaglio, T. , Parvizi, E. , Rankin, J. , Pilo, G. S. , & Velásquez, M. (2022). Giant kelp rafts wash ashore 450 km from the nearest populations and against the dominant ocean current. Ecology, 103, e3795. 35718754 10.1002/ecy.3795 PMC9787862

[ece370177-bib-0073] Macaya, E. , & Zuccarello, G. (2010). Genetic structure of the giant kelp *Macrocystis pyrifera* along the southeastern Pacific. Marine Ecology Progress Series, 420, 103–112.

[ece370177-bib-0074] MacMillan, C. (1900). Observations on *Lessonia* . Botanical Gazette, 30, 318–384.

[ece370177-bib-0116] Metzger, J. R., Konar, B., & Edwards, M. S. (2019). Assessing a macroalgal foundation species: community variation with shifting algal assemblages. Marine Biology, 166(12). 10.1007/s00227-019-3606-1

[ece370177-bib-0075] McNair, J. N. , Newbold, J. D. , & Hart, D. D. (1997). Turbulent transport of suspended particles and dispersing benthic organisms: How long to hit bottom? Journal of Theoretical Biology, 188, 29–52. 10.1006/jtbi.2001.2273 11312595

[ece370177-bib-0076] McPherson, M. L. , Finger, D. J. I. , Houskeeper, H. F. , Bell, T. W. , Carr, M. H. , Rogers‐Bennett, L. , & Kudela, R. M. (2021). Large‐scale shift in the structure of a kelp forest ecosystem co‐occurs with an epizootic and marine heatwave. Communications Biology, 4, 298. 33674760 10.1038/s42003-021-01827-6 PMC7935997

[ece370177-bib-0077] Miller, K. I. , Blain, C. O. , & Shears, N. T. (2022). Sea urchin removal as a tool for macroalgal restoration: a review on removing the “spiny enemies”. Frontiers in Marine Science, 9, 831001.

[ece370177-bib-0078] Miller, R. J. , Reed, D. C. , & Brzezinski, M. A. (2011). Partitioning of primary production among giant kelp (*Macrocystis pyrifera*), understory macroalgae, and phytoplankton on a temperate reef. Limnology and Oceanography, 56, 119–132.

[ece370177-bib-0079] North, E. (1971). In J. Wheeler (Ed.), The biology of Giant Kelp beds (Macrocystis) in California. Schweizerbart Science Publishers.

[ece370177-bib-0080] Norton, T. A. , & Fetter, R. (1981). The settlement of *Sargassum muticum* propagules in stationary and flowing water. Journal of the Marine Biological Association of the United Kingdom, 61, 929–940.

[ece370177-bib-0081] Raimondi, P. T. , Reed, D. C. , Gaylord, B. , & Washburn, L. (2004). Effects of self‐fertilization in the giant kelp, *Macrocystis pyrifera* . Ecology, 85, 3267–3276.

[ece370177-bib-0082] Reed, D. , Washburn, L. , Rassweiler, A. , Miller, R. , Bell, T. , & Harrer, S. (2016). Extreme warming challenges sentinel status of kelp forests as indicators of climate change. Nature Communications, 7, 13757. 10.1038/ncomms13757 PMC5159872 27958273

[ece370177-bib-0083] Reed, D. C. , Anderson, T. W. , Ebeling, A. W. , & Anghera, M. (1997). The role of reproductive synchrony in the colonization potential of kelp. Ecology, 78, 2443–2457.

[ece370177-bib-0084] Reed, D. C. , Kinlan, B. P. , Raimondi, P. T. , Washburn, L. , Gaylord, B. , & Drake, P. T. (2006). A metapopulation perspective on the patch dynamics of Giant Kelp in Southern California. Marine Metapopulations. In Marine Metapopulations (pp. 353–386). Elsevier.

[ece370177-bib-0085] Reed, D. C. , Laur, D. R. , & Ebeling, A. W. (1988). Variation in algal dispersal and recruitment: The importance of episodic events. Ecological Monographs, 58, 321–335.

[ece370177-bib-0086] Reed, D. C. , Neushul, M. , & Ebeling, A. W. (1991). Role of settlement density on gametophyte growth and reproduction in the kelps *Pterygophora california* and *Macrocystis pyrifera* (Phaeophyceae). Journal of Phycology, 27, 361–366.

[ece370177-bib-0087] Rogers‐Bennett, L. , & Catton, C. A. (2019). Marine heat wave and multiple stressors tip bull kelp forest to sea urchin barrens. Scientific Reports, 9, 15050. 31636286 10.1038/s41598-019-51114-y PMC6803666

[ece370177-bib-0088] Ronce, O. (2007). How does it feel to be like a rolling stone? Ten questions about dispersal evolution. Annual Review of Ecology, Evolution, and Systematics, 38, 231–253.

[ece370177-bib-0089] Sala, E. , & Graham, M. H. (2002). Community‐wide distribution of predator–prey interaction strength in kelp forests. Proceedings of the National Academy of Sciences of the United States of America, 99, 3678–3683. 11891292 10.1073/pnas.052028499 PMC122583

[ece370177-bib-0090] Santelices, B. (1990). Patterns of reproduction, dispersal and recruitment in seaweeds. Oceanography and Marine Biology: An Annual Review, 28, 177–276.

[ece370177-bib-0092] Scagel, R. F. (1947). *An investigation on marine plants near Hardy Bay*, *B.C. Victoria*, *British Columbia*, *Canada* .

[ece370177-bib-0093] Scheibling, R. E. , Hennigar, A. W. , & Balch, T. (1999). Destructive grazing, epiphytism, and disease: The dynamics of sea urchin – kelp interactions in Nova Scotia. Canadian Journal of Fisheries and Aquatic Sciences, 56, 2300–2314.

[ece370177-bib-0094] Schiel, D. R. , & Foster, M. S. (2006). The population biology of large brown seaweeds: Ecological consequences of multiphase life histories in dynamic coastal environments. Annual Review of Ecology, Evolution, and Systematics, 37, 343–372.

[ece370177-bib-0095] Schiel, D. R. , & Foster, M. S. (2015). The biology and ecology of Giant Kelp forests. University of California Press.

[ece370177-bib-0096] Schleicher, A. , Biedermann, R. , & Kleyer, M. (2011). Dispersal traits determine plant response to habitat connectivity in an urban landscape. Landscape Ecology, 26, 529–540.

[ece370177-bib-0115] Schindler, D. E., Armstrong, J. B., & Reed, T. E. (2015). The portfolio concept in ecology and evolution. Frontiers in Ecology and the Environment, 13(5), 257–263. 10.1890/140275

[ece370177-bib-0097] Seymour, R. J. , Tegner, M. J. , Dayton, P. K. , & Parnell, P. E. (1989). Storm wave induced mortality of giant kelp, *Macrocystis pyrifera*, in Southern California. Estuarine, Coastal and Shelf Science, 28, 277–292.

[ece370177-bib-0098] Smale, D. A. (2020). Impacts of ocean warming on kelp forest ecosystems. New Phytologist, 225, 1447–1454. 31400287 10.1111/nph.16107

[ece370177-bib-0099] Spector, M. , & Edwards, M. S. (2020). Species‐specific biomass drives macroalgal benthic primary production on temperate rocky reefs. Algae, 35, 237–252.

[ece370177-bib-0100] Tegner, M. J. , Dayton, P. K. , Edwards, P. B. , & Riser, K. L. (1995). Sea urchin cavitation of giant kelp (*Macrocystis pyrifera* C. Agardh) holdfasts and its effects on kelp mortality across a large California forest. Journal of Experimental Marine Biology and Ecology, 191, 83–99.

[ece370177-bib-0101] Thiel, M. (2003). Rafting of benthic macrofauna: Important factors determining the temporal succession of the assemblage on detached macroalgae. Hydrobiologia, 503, 49–57.

[ece370177-bib-0102] Underwood, J. N. , Smith, L. D. , Van Oppen, M. J. H. , & Gilmour, J. P. (2006). Multiple scales of genetic connectivity in a brooding coral on isolated reefs following catastrophic bleaching. Molecular Ecology, 16, 771–784. 10.1111/j.1365-294X.2006.03187.x 17284210

[ece370177-bib-0103] van den Hoek, C. (1987). The possible significance of long‐range dispersal for the biogeography of seaweeds. Helgoländer Meeresuntersuchungen, 41, 261–272.

[ece370177-bib-0104] VanMeter, K. , & Edwards, M. S. (2013). The effects of mysid grazing on kelp zoospore survival and settlement. Journal of Phycology, 49, 896–901. 27007314 10.1111/jpy.12100

[ece370177-bib-0105] Veenhof, R. J. , Champion, C. , Dworjanyn, S. A. , Wernberg, T. , Minne, A. J. P. , Layton, C. , Bolton, J. J. , Reed, D. C. , & Coleman, M. A. (2022). Kelp gametophytes in changing oceans. In Oceanography and marine biology: An annual review (pp. 335–371). CRC Press.

[ece370177-bib-0106] Veenhof, R. J. , Coleman, M. A. , Champion, C. , & Dworjanyn, S. A. (2023). Urchin grazing of kelp gametophytes in warming oceans. Journal of Phycology, 59, 838–855. 37432133 10.1111/jpy.13364

[ece370177-bib-0107] Veenhof, R. J. , Dworjanyn, S. A. , Champion, C. , & Coleman, M. A. (2022). Grazing and recovery of kelp gametophytes under ocean warming. Frontiers in Marine Science, 9, 866136.

[ece370177-bib-0108] Vogel, S. (1996). Life in moving fluids: The physical biology of flow. Princeton University Press. 467 pp.

[ece370177-bib-0109] Walker, D. C. (1980). *Sorus abscission from laminae of* Nereocystis luetkeana *(Mert.) Post. and Rup* . PhD dissertation, University of British Columbia.

[ece370177-bib-0110] Wernberg, T. , Bennett, S. , Babcock, R. C. , de Bettignies, T. , Cure, K. , Depczynski, M. , Dufois, F. , Fromont, J. , Fulton, C. J. , Hovey, R. K. , Harvey, E. S. , Holmes, T. H. , Kendrick, G. A. , Radford, B. , Santana‐Garcon, J. , Saunders, B. J. , Smale, D. A. , Thomsen, M. S. , Tuckett, C. A. , … Wilson, S. (2016). Climate‐driven regime shift of a temperate marine ecosystem. Science, 353, 169–172. 27387951 10.1126/science.aad8745

[ece370177-bib-0111] Wernberg, T. , Coleman, M. A. , Bennett, S. , Thomsen, M. S. , Tuya, F. , & Kelaher, B. P. (2018). Genetic diversity and kelp forest vulnerability to climatic stress. Scientific Reports, 8, 1851. 29382916 10.1038/s41598-018-20009-9 PMC5790012

[ece370177-bib-0112] Wernberg, T. , & Filbee‐Dexter, K. (2018). Grazers extend blue carbon transfer by slowing sinking speeds of kelp detritus. Scientific Reports, 8, 17180. 30464260 10.1038/s41598-018-34721-z PMC6249265

[ece370177-bib-0113] Wiberg, P. , & Smith, J. D. (1983). A comparison of field data and theoretical models for wave‐current interactions at the bed on the continental shelf. Continental Shelf Research, 2, 147–162.

[ece370177-bib-0114] ZoBell, C. E. (1971). Drift seaweeds on San Diego County beaches. In W. J. North (Ed.), The biology of giant kelp beds (Macrocystis) in California (pp. 269–314). Verlag Von J. Cramer.

